# Indicators related to smoking cessation in Brazil, National Health Survey, 2013 and 2019 editions

**DOI:** 10.1590/SS2237-9622202200005.especial

**Published:** 2022-06-29

**Authors:** Patrícia Pereira Vasconcelos de Oliveira, Vinícius Oliveira de Moura Pereira, Sheila Rizzato Stopa, Paula Carvalho de Freitas, André Salem Szklo, Tânia Maria Cavalcante, Fabiana Martins Dias de Andrade, Crizian Saar Gomes, Deborah Carvalho Malta

**Affiliations:** 1 Ministério da Saúde, Secretaria de Vigilância em Saúde, Brasília, DF, Brazil; 2 Instituto Nacional de Câncer José de Alencar Gomes da Silva, Divisão de Pesquisa Populacional, Rio de Janeiro, RJ, Brazil; 3 Instituto Nacional de Câncer José de Alencar Gomes da Silva, Secretaria Executiva da Comissão Nacional para a Implementação da Convenção-Quadro para o Controle do Tabaco, Rio de Janeiro, RJ, Brazil; 4 Universidade Federal de Minas Gerais, Programa de Pós-Graduação em Saúde Pública, Belo Horizonte, MG, Brazil; 5 Universidade Federal de Minas Gerais, Departamento de Enfermagem Materno-Infantil e Saúde Pública, Belo Horizonte, MG, Brazil

**Keywords:** Tobacco Use Cessation, Smokers, Ex-Smokers, Health Surveys

## Abstract

**Objective:**

To describe the indicators of smoking cessation in 2013 and 2019 for Brazil and federative units, according to sociodemographic variables, collected in the National Health Survey (PNS).

**Methods:**

Cross-sectional, population-based and descriptive study with data from the 2013 and 2019 PNS, a household survey collected by trained interviewers. The prevalence of ex-smokers and the proportion of smokers who tried to quit smoking in the 12 months prior to the interview, and respective confidence intervals (95%CI) were calculated, according to sociodemographic variables. Additionally, the percentage variation between the years was calculated.

**Results:**

In 2013, the prevalence of ex-smokers was 17.5% (95%CI 16.9;18.0) and, in 2019, 26.6% (95%CI 26.1;27.2). In 2013, 51.1% tried to quit smoking (95%CI 49.3;52.9) and, in 2019, 46.6% (95%CI 45.0;48.3).

**Conclusion:**

It is important to strengthen and maintain strategies for coping with tobacco use in Brazil, to increase the current smoker's willingness and ability to quit smoking.

**Study contributions**

**Main results**

In 2013, the proportion of former smokers was 17.5% (95%CI 16.9;18.0), and, in 2019, 26.6% (95%CI 26.1;27.2), which represents a 52% increase. In 2013, 51.1% (95%CI 49.3;52.9) attempted to quit smoking, and, in 2019, 46.6% (95%CI 45.0;48.3), which indicates an 8.8% reduction in the period

**Implications for services**

The study brings a reflection on the reduction in the demand for cessation treatment, an important component of the National Policy on Tobacco Control (PNCT), offered by the Brazilian National Health System (SUS). Such an effect may result in the reduction of the rate of decline in the prevalence of smokers.

**Perspectives**

It is necessary to make progress in the maintenance of actions and strategies to combat tobacco use, such as the readjustment of the taxes and the minimum price of tobacco products, intensification of regulatory measures and greater involvement of the media in anti-smoking actions.

## Introduction

Smoking has been highlighted as one of the main behavioral risk factors for noncommunicable chronic diseases (NCDs), responsible for a high number of deaths worldwide.^1^ ^-^ ^3^ It is listed in the International Classification of Diseases, Tenth Revision (ICD-10) as nicotine dependence (F17.2) and, as such, it is considered a chronic disease with periods of remission and relapse.^4^ Projections of global mortality and disease burden, from 2002 to 2030, indicate that 10% of the deaths worldwide (8 million deaths per year) will be related to smoking by the year 2030.^3^ ^,^ ^5^ ^,^ ^6^ It should be noted that most of the deaths occur in low and middle income countries, which are usually the target of intense marketing and interference from the tobacco industry.^7^

In response to this serious public health problem, the Global Action Plan for the Prevention and Control of NCDs set a goal of 30% relative reduction in prevalence of tobacco use in individuals aged 15 or over, between 2015 and 2025.^6^ In the context of the Sustainable Development Goals (SDGs), the established goal was strengthening the implementation of the Framework Convention on Tobacco Control in all countries, as approriate.^8^ In the Brazilian context, it is important to highlight the 2011-2022 Strategic Action Plan to Tackle Non-communicable Diseases^6^ and the National Policy on Tobacco Control (*Política Nacional de Controle do Tabaco* - PNCT), which are important instruments to reduce smoking prevalence and morbimortality related to the consumption of tobacco products in Brazil.

As a result of national efforts, there has been significant decrease in smoking prevalence in the past decades. From 1989 to 2003, smoking among adults in Brazil was reduced by 2.5% per year in average, namely, from 34.8% to 22.4%.^9^ Data from the 2008 National Household Sample Survey (Pnad) and the 2013 National Health Survey (PNS) showed that during this period there was a 19% decline in the prevalence of tobacco smokers, from 18.2% in 2008 to 14.7% in 2013. In addition, it was observed that such decline occurred in all the regions, in both urban and rural areas, as well as in most of the states.^10^ A time series analysis, based on data from the Chronic Disease Risk and Protective Factors Surveillance Telephone Survey (Vigitel), showed that smoking prevalence presented a significant decrease of about 4% a year, ranging from 15.7% in 2006 to 9.8% in 2019. However, the decrease in intensity in recent years is worrisome and may indicate the stagnation of policies currently implemented.^1^

In order to appraise the actions for tobacco control in a population, on top of identifying smoking prevalence, it is essential to monitor the cessation of tobacco use, or other indicators that can measure the prevalence of ex-smokers.^9^ Monitoring the national data on risk factors for NCDs is a highly relevant measure in the assessment of public policies, especially in view of the global commitments made by Brazil to the World Health Organization (WHO) and the United Nations (UN), in terms of reducing the prevalence of smoking^2^ ^,^ ^10^, through the Strategic Action Plan to Tackle Non-communicable Diseases.

This study aimed to describe the indicators of smoking cessation in 2013 and 2019, for Brazil and the Federative Units (FUs), according to sociodemographic variables, collected in the PNS.

## Methods

### Study desing

This was a cross-sectional, population-based, descriptive study in which data from the 2013 and 2019 PNS, carried out by the Brazilian Institute of Geography and Statistics (IBGE), in partnership with the Ministry of Health, were analyzed.

### Data source

PNS is a household survey representative of Brazil and the population residing on Brazilian territory. This survey included individuals aged ≥ 15 years and residing in private households. The following collectives were excluded: indigenous people, barracks, military bases, lodgings and campsites, all types of prisons and long-stay institutions for the elderly, among others.^11^ ^,^ ^12^

For the 2013 PNS we used a three-stage cluster sampling design: census tracts or groups of sectors (primary units), households (secondary units) and adult residents (≥ 18 years old) (tertiary units). In 2019, the sampling plan followed the same criteria as in the 2013 edition, except for the third stage, in which a household resident was randomly selected among those aged ≥ 15 or over, based on the list of residents obtained at the moment of interview. Therefore, aiming to enable data comparability between the two editions of the PNS, this study only analyzed data pertaining to residents aged 18 years and over.^11^

To calculate the sample size, the following were considered: mean value, variances, and the effects of the sampling plan with an estimated non-response rate of 20%. In 2013, 69,994 households were occupied and 64,348 valid interviews were conducted. In 2019, 108,525 households were visited and 94,114 interviews were conducted. The non-response rates were 8.1% and 6.4%, respectively. To perform the analysis, weighting factors were considered, in order to take into account effects from the stratification and clustering process in the estimates of the indicators. The weighting factors employed were the primary sampling units, stratum and weighting.^11^ ^,^ ^13^ ^,^ ^14^

### Study variables

The study analyzed data from the selected resident, pertaining to questions about tobacco use cessation, which were similar to those used in the Global Adult Tabacco Survey, conducted in several countries and coordinated by WHO, the Centers for Disease Control and Prevention (CDC), and the Pan American Health Organization (PAHO).^15^ In Brazil, the questionnaire was applied in full in the 2008 Pnad edition, and a shortened version was used in the 2013 and 2019 PNS editions.^16^ In our study, the following indicators were privileged: i) prevalence of ex-smokers; and, ii) proportion of smokers that had made a quit attempt in the 12 months prior to the interview. For the second indicator, the term proportion was used because it was calculated from a fraction of the sample. Box 1 describes the operationalization and the calculations of the indicators analyzed.

**Box 1:** Operationalization and calculation method of the study’s indicators, National Health Survey, 2013 and 2019, Brazil

Indicators	Questions 2013	Questions 2019	Calculation method
Prevalence (%) of former tobacco smokers aged 18 or over.	P50. *Do you currently smoke any tobacco product?*, and response option 3 “I don’t currently smoke” was considered; and	P50. *Do you currently smoke any tobacco product?*, and response option 3 “I don’t currently smoke” was considered; and	Number of ex-smokers (P50 = 3 and P52 = 1 or P52 = 2)/Number of individuals aged 18 or over who were interviewed.
	P52. *Do you currently smoke any tobacco product?*, and response options 1 or 2, “yes, daily”, and “yes, less than daily”, were considered.	P52. *Do you currently smoke any tobacco product?*, and response options 1 or 2, “yes, daily”, and “yes, less than daily”, were considered.	
Proportion (%) of tobacco smokers aged 18 or over who attempted to quit smoking in the last 12 months	P60. *Have you attempted to quit smoking over the past 12 months?*, and response option 1,”yes”, was considered; or,	P60. *Have you attempted to quit smoking over the past 12 months*?, and response option 1,”yes”, was considered; or,	Number of current tobacco smokers who had tried to quit smoking in the 12 months prior to the survey (P60 = 1 or P59 = 0 years)/Number of individuals interviewed who currently smoke or who stopped smoking less than a year ago (P50 = 1 or 2 or P59 = 0 years).
	P59. *How long has it been since you stopped smoking?*, and a response equal to 0 years was considered.	P59. *How long has it been since you stopped smoking?*, and a response equal to 0 years was considered.	

The sociodemographic characteristics studied were:

- place of residence (urban; rural);
- sex (male; female);
- age group (18 to 24; 25 to 39; 40 to 59; and ≥ 60);
- level of education (no schooling and incomplete primary education; complete primary education and incomplete secondary education; complete secondary education and incomplete higher education; complete higher education);
- race/skin color ([White; Black; Brown]; yellow and indigenous race/skin color were considered only in the total, due to the small number of observations and high coefficient of variation);
- region (North, Northeast, Southeast, South, Midwest); and
- income per capita (no income or up to 1/4 of the minimum wage [MW]; more than 1/4 to 1/2 MW; more than 1/2 to 1 MW; more than 1 MW to 2 MWs; more than 2 to 3 MWs; more than 3 to 5 MWs; more than 5 MWs).

### Data analysis

The prevalence of ex-smokers and the proportions of smokers who had attempted to quit smoking in the 12 months prior to the interview, as well as their 95% confidence intervals (95%CI) were calculated, in 2013 and 2019, for the total population and the FUs, according to sociodemographic characteristics. In addition, the percentage variation between the years studied was calculated, by subtracting the percentages obtained in 2019 and 2013.

The statistical analysis was performed using Stata software, version 14.0 (StataCorp) and, considering the survey data analysis involves complex sampling, the survey module was used to apply weighting, selection of the household resident and age group ≥ 18 years.

### Ethical aspects

Data from the PNS are available for public access and use, and both editions were approved by the National Research Ethics Committee of the National Health Council, under Opinion No. 328,159 for the 2013 edition, and No. 3,529,376 for the 2019 edition.

## Results

In 2013, 60,202 individuals aged 18 or over were interviewed and, in 2019, the number of respondents aged 18 or over was 88,531.

In 2013, the prevalence of ex-smokers was 17.5% (95%CI 16.9;18.0), while in 2019 it increased to 26.6% (95%CI 26.1;27.2); the variation between years was 52%. The prevalence of ex-smokers increased when considering the area of residence, being higher in the urban area when compared with the rural area, with an increase of 54.6% and 36.8%, respectively, in 2019. Regarding sex, in 2013 the prevalence was 21.2% (95%CI 20.3;22.1) and, in 2019, it increased to 26.8% (95%CI 26.1;27.5) for males. For females, it was 14.1% (95%CI 13.4;14.8) in 2013, and 26.5% (95%CI 25.7;27.2) in 2019. Increase in the prevalence of smoking cessation was higher for females: +87.9% *versus* +26.4% for males (Table 1).

**Table 1:** Prevalence of ex-smokers in the 12 months prior to the interview and 95% confidence intervals, according to sociodemographic variables, National Health Survey, 2013 and 2019, Brazil

Variables	Ex-smokers				Variation (%) 2013/2019
	2013 (n = 60,202)		2019 (n = 88,531)		
	%^a^	95%CI^b^	%^a^	95%CI^b^	
**Area of residence**					
Urban	17.2	16.5;17.8	26.6	26.0;27.3	54.7
Rural	19.3	17.8;20.8	26.4	25.4;27.4	36.8
**Sex**					
Male	21.2	20.3;22.1	26.8	26.1;27.5	26.4
Female	14.1	13.4;14.8	26.5	25.7;27.2	87.9
**Level of education**					
No schooling and incomplete elementary education	24.2	23.2;25.2	33.9	33.0;34.8	40.1
Complete primary education and incomplete secondary education	14.8	13.5;16.2	26.1	24.7;27.5	76.4
Complete secondary education and incomplete higher education	11.9	11.0;12.8	22.3	21.4;23.3	87.4
Complete higher education	14.4	12.9;15.8	20.7	19.6;21.8	43.8
**Age group (years)**					
18 to 24	5.6	4.8;6.5	18.6	17.0;20.2	232.1
25 to 39	11.5	10.6;-12.4	18.7	17.8;19.6	62.6
40 to 59	21.3	20.2;22.3	26.8	25.9;27.7	25.8
60 or over	31.1	29.6;-32.6	42.2	41.1;43.3	35.7
**Region**					
North	16.6	15.3;17.9	23.2	22.0;24.4	39.8
Northeast	18.1	17.1;19.0	27.0	26.2;27.8	49.2
Southeast	17.1	16.1;18.2	27.5	26.5;28.6	60.8
South	18.3	16.9;19.7	26.7	25.6;27.9	45.9
Midwest	16.3	15.2;17.5	23.5	22.2;24.7	44.2
**Race/skin color^c^**					
White	17.8	16.9;18.6	26.4	25.5;27.3	48.3
Black	16.1	14.2;-17.9	28.8	27.2;30.5	78.9
Brown	17.4	16.6;18.2	26.2	25.5;27.0	50.6
**Total**	17.5	16.9;18.0	26.6	26.1;27.2	52.0

a) %: Prevalence; b) 95%CI: 95% confidence interval; c) The other categories of race/skin color (Yellow and indigenous) were not included due to the small number of observations.

When considering education, the prevalence of ex-smokers increased in all educational strata. It was higher among those with no schooling and incomplete primary education: 24.2% (95%CI 23.2;25.2) in 2013, and 33.9% (95%CI 33.0;34.8) in 2019. However, when assessing the increase over the years, it was observed that the prevalence of ex-smokers was higher in the strata of complete secondary education and complete higher education, corresponding to an 87.4% variation. In terms of age groups, the prevalence of ex-smokers increased in all age ranges, and the percentage variation between the years studied was 232.1%, among those aged 18 to 24 years.

When analyzing the Brazilian regions, the prevalence in 2013 was lower in the Midwest and North regions, 16.3% (95%CI 15.2;17.5) and 16.6% (95%CI 15.3;17.9) respectively, and higher in the South, with 18.3% (95%CI 16.9;19.7), followed by the Southeast with 18.1% (95%CI 17.1;19.0). Analyzing data for 2019, the North region maintained the lowest prevalence, 23.2% (95%CI 22.0;24.4), and the Southeast the highest, 27.5% (95%CI 26.5;28.6). The highest percentage variation between the years was 60.8% in the Southeast, and the lowest was 39.8% in the North region (Table 1).

In terms of the proportion of adults who tried to quit smoking, in 2013 51.1% (95%CI 49.3;52.9) of the respondents reported the attempt and, in 2019, the proportion was 46.6% (95%CI 45.0;48.3), with an 8.8% variation between the years. When considering the urban area, the proportion of adults that tried to quit smoking went from 51.2% (95%CI 49.2;53.3) in 2013 to 46.7% (95%CI 44.8;48.5) in 2019. Analyzing by sex, the percentage variation was higher for females (-9.1%), as can be seen in Table 2.

**Table 2:** Proportion of quit attempts in the 12 months prior to the interview and 95% confidence intervals, according to sociodemographic variables, National Health Survey, 2013 and 2019, Brazil

Variables	Tried to quit smoking				Variation (%) 2013/2019
	2013 (n = 9,420)		2019 (n = 12,273)		
	%^a^	95%CI^b^	%^a^	95%CI^b^	
**Area of residence**					
Urban	51.2	49.2;53.3	46.7	44.8;48.5	-8.8
Rural	50.3	47.0;53.6	46.4	43.3;49.5	-7.8
**Sex**					
Male	47.9	45.5;50.4	43.8	41.6;46.0	-8.6
Female	55.9	53.3;58.6	50.8	48.5;53.2	-9.1
**Level of education**					
No schooling and incomplete elementary education	51.6	49.2;54.0	47.4	45.1;49.6	-8.1
Complete primary education and incomplete secondary education	52.4	47.5;57.3	51.1	47.0;55.1	-2.5
Complete secondary education and incomplete higher education	51.1	47.1;55.0	44.5	41.3;47.8	-12.9
Complete higher education	44.8	38.6;51.0	40.5	35.3;45.7	-9.6
**Age group (years)**					
18 to 24	54.0	48.1;60.0	51.5	45.8;57.2	-4.6
25 to 39	53.7	50.4;56.9	48.0	45.0;51.1	-10.6
40 to 59	50.4	47.7;53.1	44.9	42.4;47.5	-10.9
60 or over	45.9	41.3;50.4	45.1	42.1;48.1	-1.7
**Region**					
North	49.9	46.4;53.5	46.5	42.7;50.3	-6.8
Northeast	53.7	50.5;57.0	50.0	47.2;52.7	-6.9
Southeast	49.7	46.5;52.9	45.9	42.9;48.8	-7.6
South	52.1	48.3;55.9	44.3	41.1;47.6	-15.0
Midwest	48.7	44.6;52.8	46.6	42.8;50.4	-4.3
**Race/skin color^c^**					
White	49.0	46.1;51.9	42.8	40.1;45.4	-12.7
Black	49.9	44.0;55.9	54.1	49.9;58.2	8.4
Brown	53.0	50.4;55.7	48.2	45.9;50.5	-9.1
**Total**	51.1	49.3;52.9	46.6	45.0;48.3	-8.8

a) %: Proportion; b) 95%CI: 95% confidence interval; c) The other categories of race/skin color (Yellow and indigenous) were not included due to the small number of observations.

For education, the proportions remained stable. However, despite the confidence intervals overlap, specific proportions suggest a decreasing trend, especially in the group with the lowest educational level and in the group with complete secondary education and incomplete higher education. When considering age, there was a decrease in smoking cessation attempts only in the 40 to 59 age group (10.9%). For the regions, there was a reduction in the indicator of the South region, with a 15% variation. With regard to race/skin color, a reduction in attempts to quit smoking was observed only among white individuals (Table 2).

**Figure 1:**
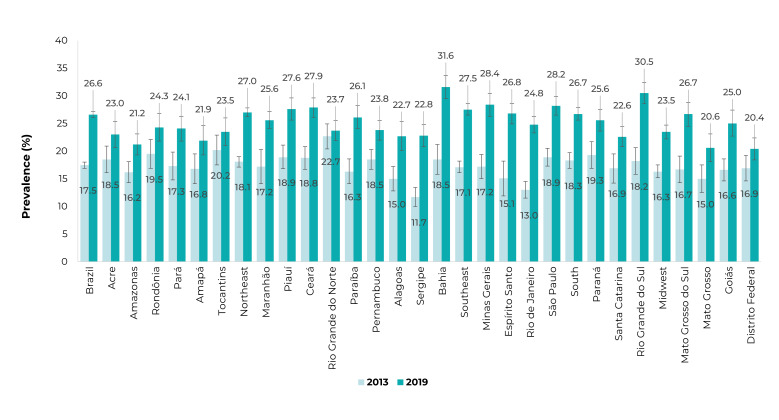
Prevalence of former tobacco smokers in the 12 months prior to the interview, in Brazil and federative units, National Health Survey, 2013 and 2019, Brazil

**Figure 2:**
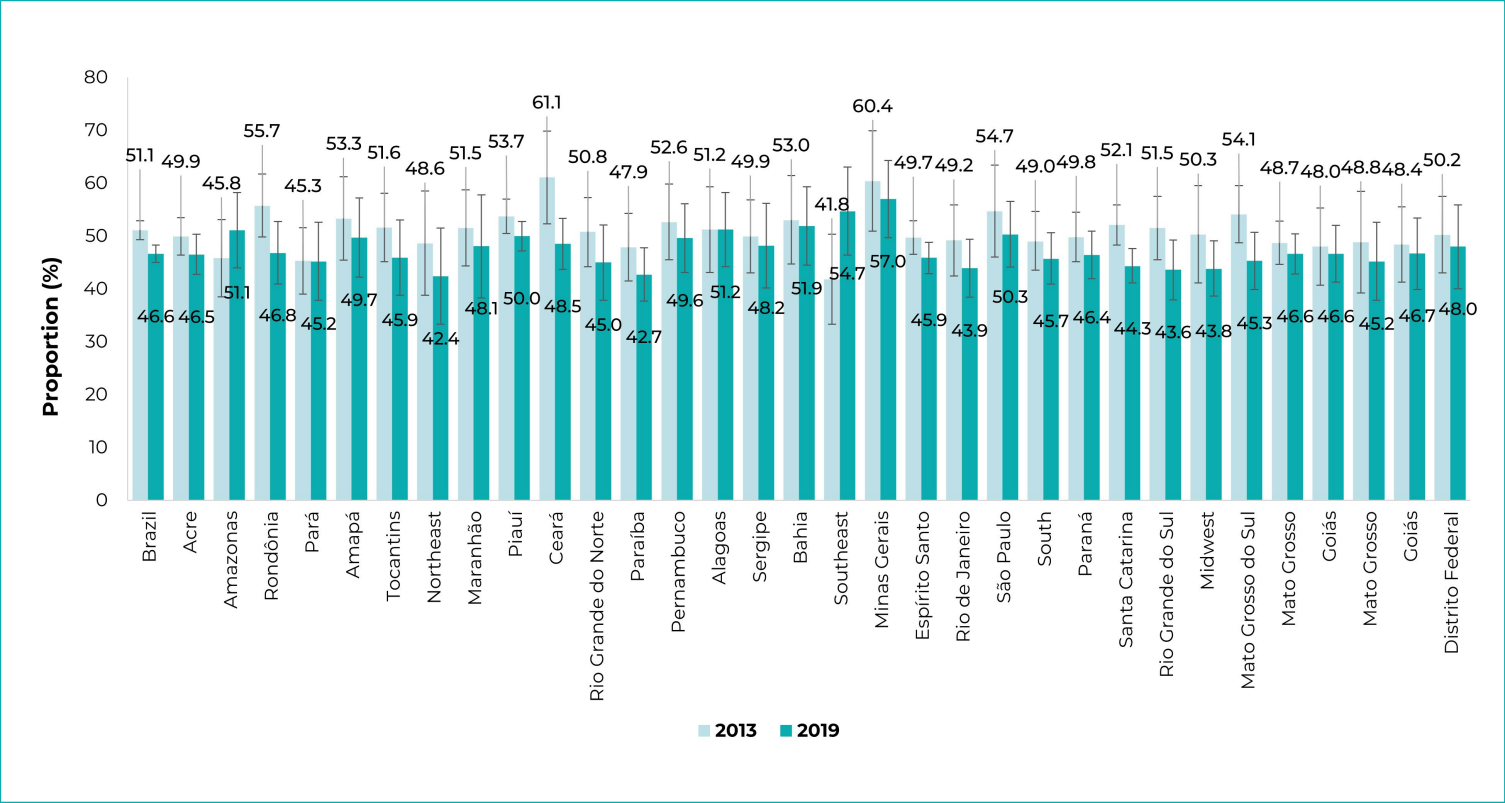
Proportion of tobacco smokers who attempted to quit smoking in the 12 months prior to the interview, in Brazil and federative units, National Health Survey, 2013 and 2019, Brazil

Figure 1 presents the distribution of ex-smokers according to the FUs. The highest proportions of ex-smokers in 2013 were found in Rio Grande do Norte, Tocantins, and Rondônia. In 2019, on the other hand, the highest proportions were found in Bahia, Rio Grande do Sul, Minas Gerais, and São Paulo (Figure 1). Figure 2 shows the distribution of people who tried to quit smoking by FUs. In 2013, the highest proportions of individuals who tried to quit smoking were found in Maranhão and in Bahia, and in 2019, also in Bahia and in Sergipe.

## Discussion

This study identified a change in the indicators of tobacco use cessation between the years 2013 and 2019, with an increase of ex-smokers in terms of the total population and when considering all sociodemographic strata. On the other hand, the results suggest that there has been a decrease in smoking cessation attempts, even though the prevalence have remained stable for most of the strata studied, from the perspective of the 95%CI overlap.

Smoking cessation support is one of the components of the National Policy on Tobacco Control, in accordance with article 14 of the Framework Convention on Tobacco Control.^17^ Smoking cessation treatment, also known as nicotine dependence treatment, has been offered free of charge by the Brazilian National Health System (SUS) since 2004, being an important measure for the reduction of tobacco demand and, therefore, a fundamental action to ensure the global target is met.^17^ This measure is reinforced by the offer of counseling for smoking cessation through the Dial Health (Disque Saúde) service, which is mandatorily displayed along with the health warning labels on cigarette packs.^18^ With the implementation of smoke-free laws, public policies and the offer of smoking cessation treatment, in addition to strengthening the Framework Convention on Tobacco Control, Brazil became, in 2019, an international reference in tobacco control, reaching the highest level of the MPOWER package of strategies, based on the framework, and an integral part of the WHO Action Plan for the Prevention and Control of Non-communicable Diseases.

The MPOWER measures are: monitoring tobacco use and prevention policies; protecting people from tobacco smoke; offering support for smoking cessation; warning about the dangers of tobacco; enforcing tobacco advertising, promotion and sponsorship bans, and raising taxes on tobacco products.^19^ However, smoking cessation still poses a great challenge for public health.^17^

Smoking cessation treatment is recognized as one of the most cost-effective clinical interventions when compared with the treatment of diseases caused by smoking.^20^ However, reaching the population is still a major challenge in comparison with the other measures of the National Policy, considering that this is the only measure that has an individual coverage, therefore, the one that depends on greater investment for the treatment coverage to be expanded, achieved through the acquisition of medication and qualified health professional teams. Thus, in order to expand its reach, WHO has been taking actions to ensure that countries invest in interventions that encourage and support smoking cessation through mobile applications, focusing on the population in the lower income and education strata.^21^

Success in quitting smoking depends on several factors. However, two of the most important ones are related to the level of nicotine dependence and the smoker’s motivation to stop smoking.^22^ Evidence shows that brief counseling by a health professional and behavioral support are effective in motivating smokers to quit, and that more intensive interventions are more effective in motivating and helping smokers to quit in comparison with minimal interventions, mainly for those with higher levels of nicotine dependence, according to the Fagerström Test. Protocols and recommendations that guide clinicians and health systems regarding counseling and the use of medication to treat nicotine dependence argue that smoking be recognized as a chronic disease that requires repeated intervention and multiple attempts for cessation, which points to the need of significant investment in such action.^23^

The actions to diminish tobacco use in the past decade have been important for the reduction of smoking prevalence in Brazil. The present study revealed that there has been an increase in ex-smokers, especially in the urban area, in contrast to the rural area, and among the younger population. It can be inferred that this scenario took place due to actions such as smoke-free laws, which are more intensively enforced in urban areas. Another important factor to be taken into consideration is the change in relation to the social acceptability of smoking, which evolved to a context of rejection in the 2000s, which might have influenced the increased number of ex-smokers, especially among younger people.^17^

Another effective action that promotes the reduction in prevalence and experimentation with tobacco is the increase in taxes and retail sales price of tobacco products. It is worth noting that since 2015 the country has been going through an economic crisis, which has worsened in recent years, which was probably a contributing factor to the increased demand for treatment. Spending on tobacco can compromise between 4.8% to 7% of family expenses. With such a crisis, it is assumed that families had to reduce their spending, including the purchase of cigarettes.^24^

In Brazil, successive adjustments of tobacco excise tax rates have been adopted since 2007.^25^ Studies from the World Bank have shown that populations with lower incomes and education, as well as young people, are the most susceptible to increase in prices.^7^ Therefore, it is possible that the higher prevalence of ex-smokers in the group with lower level of education observed in 2019, when compared with 2013, is also a consequence of price increases, due to rises in the Tax on Industrialized Products (IPI), starting in 2012, in addition to the creation of a minimum price policy, whose level increased incrementally every year, until 2016.^26^ It is important to highlight that, since 2016, both the IPI and the minimum price have not been readjusted, and the real price of a cigarette pack has been decreasing since 2017. As the present paper covers a broad period, from 2013 to 2019, during which the price and tax policy underwent advances and setbacks, it is reasonable to consider that the result obtained in the prevalence of smoke cessation could have been even higher, if the policy had not stagnated in 2016.

It is worth highlighting that the increased proportion of ex-smokers in all geographic, sociodemographic and economic strata points to the immense contribution of SUS in generating healthcare comprehensiveness and reducing health inequalities. Despite the increase in the number of ex-smokers and search for treatment, the emergence of new forms of tobacco use in the last decade poses a challenge to the advancement of tobacco use cessation.^27^

In spite of the reduction in the prevalence of smokers between 2013 and 2019, it was lower in comparison with 2008. There still are over 20 million smokers in Brazil, which represents a great challenge when ensuring access to smoking cessation treatment. However, it is worth noting that the cost of treating diseases caused by smoking far exceed the cost of treatment for nicotine dependence.^28^

Data from Vigitel survey on tobacco use prevalence in Brazil confirm the PNS results and highlight the reduction in the pace of decline of tobacco use after 2016. This behavior might be the effect of the weakening of the regulatory measures that had been implemented in the country over the years, and it is necessary to reinstitute more effective strategies, including raising the price of tobacco products.^29^

The present study adds a reflection on what the observed reduction in the proportion of attempts to quit smoking in the last 12 months, either on their own initiative or through seeking specialized help, between the periods studied, could mean. Unlike the cumulative increase in former smokers identified between 2013 and 2019, a lower willingness of current smokers to quit over the year prior to the survey period probably reflects the negative impacts of the recent weakening of the main measure to reduce the prevalence of smokers: taxation measures. In fact, between 2008 and 2013, shortly after the tax reform implemented in 2012, which substantially increased the real price of tobacco products, Brazil registered a significant increase in the prevalence of smokers who reported trying to quit smoking in the last 12 months, either by sex, schooling, age group or area of residence.^30^ Considering that the more the smoker tries to quit smoking, the greater the probability that he will manage to become a former smoker, the findings of the present study are a warning signal that Brazil needs to continue advancing in the implementation of effective measures to control tobacco use.

In this sense, investing in actions that reinforce the harmful effects of smoking, such as social communication and media presence, are of utmost importance.^12^ Therefore, this study highlights the importance of maintaining continuity of public policies to combat tobacco use. It also necessary that the Framework Convention on Tobacco Control measures by applied to new products that are gaining market, such as electronic cigarettes and hookhas, and that regulations advance so that the strong tobacco industry lobbying can be combatted. Electronic cigarettes, very popular among young people, advertised and promoted by the tobacco industry as being low risk, or even risk free, can stimulate this population to reduce quit attempts. On top of that, such devices have been used, with no evidence, for tobacco use cessation, replacing classic smoking. Considering that electronic cigarettes are used for a short period of time, this behavior is a source of concern and there is the risk of the smoker falling back into classic smoking.^27^

Among the limitations of this study, the cross-sectional design should be mentioned, which makes it difficult to establish a temporal relationship between the events and to investigate whether there is a causal relationship between them. Another important aspect is the use of self-reported information to estimate the prevalence of tobacco use cessation and, therefore, the chance of underestimated results. Self-reported information might be subjected to information bias, which can cause deviations in the results obtained. Despite these limitations, taking into consideration that this study uses databases of a sample representative of the Brazilian population and with methodological rigor, and that this type of study presents lower costs and data collection is faster compared with other types of studies, its results are instrumental in supporting public health planning, contributing to understand the health-disease process and identifying hypothesis that can be studied subsequently. In addition, PNS is considered the gold standard among the population surveys conducted in Brazil. 

In conclusion, the study demonstrated that there was an increase of approximately 50% in the prevalence of ex-smokers in Brazil and a reduction of around 9% in the proportion of quit attempts in the last year, when comparing data from the 2013 and 2019 PNS. In this regard, it is important to stress the maintenance and development of actions, strategies and public policies to combat the use of tobacco in Brazil.
